# MicroRNA-140-5p represses chondrocyte pyroptosis and relieves cartilage injury in osteoarthritis by inhibiting cathepsin B/Nod-like receptor protein 3

**DOI:** 10.1080/21655979.2021.1985342

**Published:** 2021-11-29

**Authors:** Lei Zhang, Jianjun Qiu, Jixiang Shi, Shaoyang Liu, Hanlin Zou

**Affiliations:** Department of Orthopedics, Putuo Hospital, Shanghai University of Traditional Chinese Medicine, Shanghai, China

**Keywords:** Osteoarthritis, Cartilage injury, pyroptosis, miR-140-5p, ctsb, nlrp3, co-immunoprecipitation

## Abstract

Osteoarthritis (OA) is a degenerative joint disease. Dysregulated microRNA (miRNA) expressions are implicated in OA progression. Consequently, the current study set out to investigate the mechanism of miR-140-5p in OA cartilage injury. Firstly, the murine and cell models of OA were established, and cartilage tissues of OA mice were observed using hematoxylin and eosin staining and safranin O staining. Chondrocyte pyroptosis was further assessed using immunohistochemical and Calcein-AM/PI staining. The levels of gasdermin-D (GSDMD)-N, cleaved caspase-1, interleukin (IL)-1β, and IL-18 in cartilage tissues and cells were determined using Western blot and enzyme-linked immunosorbent assay kits. The targeting relationship between miR-140-5p and cathepsin B (CTSB) was verified using a dual-luciferase assay. Moreover, the binding of CTSB and Nod-like receptor protein 3 (NLRP3) was detected using co-immunoprecipitation assay. Lastly, the effects of NLRP3 activation and CTSB overexpression on chondrocyte pyroptosis were documented. It was found that OA induction aggravated cartilage tissue injury and enhanced chondrocyte pyroptosis. miR-140-5p was poorly-expressed in OA models, and miR-140-5p over-expression alleviated chondrocyte pyroptosis, as evidenced by decreased GSDMD-N, cleaved caspase-1, IL-1β, and IL-18 levels. miR-140-5p targeted the CTSB gene, whereas CTSB further bound to NLRP3 and activated the NLRP3 inflammasome. Additionally, CTSB over-expression or NLRP3 activation reversed the inhibitory effect of miR-140-5p on chondrocyte pyroptosis. Collectively, our findings revealed that miR-140-5p repressed chondrocyte pyroptosis and alleviated OA cartilage injury *via* inhibition of the CTSB/NLRP3. This study may confer a theoretical basis for the treatment of OA cartilage injury.

## 1. Introduction

Osteoarthritis (OA) is regarded the most prevalent chronic degenerative disorder in elderly populations, featured by progressive articular cartilage destruction which can lead to severe physical pain and even disability [1]. This disease can involve almost every joint in the body, accompanied by typical symptoms of joint pain and disordered articular functions [2]. The pathological changes in OA include articular cartilage degradation, subchondral bone sclerosis, synovial inflammation, osteophyte formation, and hypertrophy of the whole joint capsule [3]. The articular cartilage is a highly-specialized tissue comprising of extracellular matrix synthesized by the sparsely-distributed resident cells, chondrocytes [1]. Inherently, articular chondrocytes possess the ability to mediate the homeostasis of articular cartilage by maintaining cellular survival function and governing the balance between anabolic and catabolic functions [4]. Furthermore, the survival of chondrocytes is imperative for the synthesis and secretion of extracellular matrix, such that chondrocyte death can bring about detrimental changes in the structure of articular cartilage [1]. On a similar note, the process of pyroptosis is a form of programmed cell death triggered by inflammasomes, and previous studies have further implicated inflammasome-mediated pyroptosis in the pathogenesis of OA [5]. Moreover, another prior investigation has illustrated that the damaged chondrocytes associated with OA exhibit the morphological alternations consistent with pyroptosis, which underscores the contribution of chondrocyte pyroptosis to the pathology of OA [2]. Hence, it can be highly-beneficial to explore potent target to restrain the chondrocyte pyroptosis for the management of cartilage injury in OA.

Another topic of great interest over the last decade, microRNAs (miRNAs), as a group of small noncoding RNA molecules consisting of 18–23 nucleotides, are capable of negatively-regulating gene expression and playing vital roles in chondrocyte development and cartilage homeostasis [3]. miRNAs have been documented to exhibit tissue-specific or developmental stage-specific expression profiles associated with join diseases, such as joint disorders, rheumatoid arthritis, and OA [6]. One such miRNA, namely miR-140-5p, has been recently identified as a cartilage-specific miRNA that modulates cartilage homeostasis, and deficiency of miR-140 is associated with the development of OA-like changes [7]. In addition, miR-140-5p is highlighted to serve as a biomarker in the diagnosis and clinical management of OA, such that dysregulation of miR-140-5p in synovial fluid is correlated with OA severity [8]. Furthermore, accumulating studies have evidenced that miR-140-5p contributes to the attenuation of inflammation in OA [9–11]. For instance, up-regulation of miR-140-5p exerts a suppressive effect on the expressions of inflammation-related factors, such as interleukin (IL)-1β and tumor necrosis factor α (TNF-α) in OA synovial tissues and synoviocytes [11]. Besides, over-expression of miR-140-5p is known to alleviate inflammatory responses, matrix metalloprotease expression, and chondrocyte apoptosis [9]. What’s more, a recent study has further suggested the use of miR-140-5p for therapeutic purpose in OA due to its ability to repress inflammation and trigger chondrogenesis [12]. Meanwhile, Cathepsin B (CTSB) is known as a lysosomal cysteine protease, which serves as a marker of dedifferentiated chondrocyte phenotype, resulting in cartilage degeneration in OA [13]. CTSB activity is associated with OA severity and joint inflammation, wherein the increase of CTSB activity can stimulate the secretion of pro-inflammatory cytokines TNF-α and IL-1β [14]. Thereafter, we speculated whether miR-140-5p can mediate the expression of CTSB to participate in OA development. Considering the crucial involvement of chondrocyte pyroptosis in OA pathogenesis, we set out to investigate the effect and mechanism of miR-140-5p in chondrocyte pyroptosis, hoping to uncover a novel therapeutic target for OA cartilage injury repair.

## 2. Materials and methods

### 2.1 Ethics statement

The current study was performed following the approval from the Ethical Committee of Putuo Hospital. All animal experimentation protocols conformed to the *Guide for the care and use of laboratory animals*. Extensive efforts were made to minimize the number and suffering of the included animals.

### 2.2 The murine model establishment and grouping

Male BALB/c mice (aged 4–6 weeks; weighing 18–22 g) procured from Southern Medical University (Guangzhou, Guangdong, China) [SCXK (Guangdong) 2016–0041] were raised in a specific pathogen-free grade animal room at 18–22°C, with 50–60% humidity and maintained under a 12 h light/dark cycle. The mice were allowed *ad libitum* access to food and water.

agomiR-140-5p and agomiR-negative control (NC) (GenScript, Nanjing, Jiangsu, China) were injected into the knee-joint of mice 24 h prior to OA modeling, respectively.

Subsequently, the mice were intraperitoneally anesthetized with 1% pentobarbital (50 mg/kg) (the mice were quiet, unresponsive to external stimuli, and maintained constant heartbeat and respiratory rate). Each mouse was operated on to induce OA according to the method employed by *Glasson et al*. [15]. Briefly, after the skin of mouse was disinfected and incised, the joint capsule was opened, and then the medial meniscotibial ligament (MMTL) was transected and sutured. Sham operation was performed in parallel, with the exception of MMTL transection. Later, mice from all groups were sacrificed with intraperitoneal injection of 1% pentobarbital (200 mg/kg) 60 days after OA modeling, and the cartilage tissues of knee-joints of mice in each group were harvested for histological scoring and protein extraction. The mice were assigned into the normal group (mice without any operation), the sham group (mice that underwent all operations except the transection of MMTL), the OA group (mice that underwent OA induction operation), the OA + agomiR-NC group (mice that received agomiR-NC injection and OA induction operation), and the OA + agomiR-140-5p group (mice that received agomiR-140-5p injection and OA induction operation), with 12 mice in each group, such that 6 mice were used for histological analysis and 6 mice were used for protein detection.

### 2.3 The cell model of OA and transfection and grouping

Mouse chondrocytes were isolated by referring to the methods in previous literature [16] as follows: the articular cartilage was separated from knee-joint and detached with 0.25% trypsin ethylene diamine tetra acetic acid solution for 2 h and 0.1% collagenase II solution, overnight. Subsequently, the isolated chondrocytes were centrifuged, resuspended, and cultured in Dulbecco’s modified Eagle’s medium (DMEM)/F-12 medium containing 10% fetal bovine serum and 1% penicillin-streptomycin at 37°C with 5% CO_2_ in air. Upon reaching 80–90% confluence, the cells were passaged with trypsin solution.

Afterward, the chondrocytes at the logarithmic growth phase were seeded in 6-well plates (2 × 10^5^ cells/well). Following 24-h incubation, the cells were transfected with mimic NC, miR-140-5p mimic, pcDNA3.1-NC, or pcDNA3.1-CTSB (Genechem, Shanghai, China) (miRNA-mimic 20 nM; pcDNA3.1 10 nM) in line with the instructions of Lipofectamine 2000 kits (11,668–019, Invitrogen, Carlsbad, CA, USA). For Nod-like receptor protein 3 (NLRP3) activation, the cells were subjected to treatment with NLRP3 agonist Nigericin sodium salt (NSS) (1 µM; dimethyl sulfoxide (DMSO) as solvent) for 24 h. Subsequent experiments were performed 48 h after the transfection. Cell models of OA were induced with the help of lipopolysaccharide (LPS) (5 µg/mL; Sigma-Aldrich, Merck KGaA, Darmstadt, Germany) at 37°C for 12 h. The cells were assigned into the blank group (untreated primary chondrocytes), the OA group (primary chondrocytes subjected to OA induction), the OA + miR-NC group (primary chondrocytes subjected to mimic NC transfection and then OA induction), the OA + miR-140-5p group (primary chondrocytes subjected to miR-140-5p mimic transfection and OA induction), the OA + miR-140-5p + pc3.1-NC group (primary chondrocytes subjected to miR-140-5p mimic and pc3.1DNA-NC transfection and OA induction), the OA + miR-140-5p + pc3.1-CTSB group (primary chondrocytes subjected to miR-140-5p mimic and pc3.1DNA-CTSB transfection and OA induction), the OA + miR-140-5p + DMSO group (primary chondrocytes subjected to miR-140-5p mimic transfection, DMSO treatment, and OA induction), and the OA + miR-140-5p + NSS group (primary chondrocytes subjected to miR-140-5p mimic transfection, NSS treatment, and OA induction).

### 2.4 Histological evaluation

The knee-joint of mouse was fixed, paraffin-embedded, and sectioned (5 µm). Next, the cartilage surface and cell morphology were observed by means of hematoxylin and eosin staining (HE staining). The cartilage tissue was further stained with 0.04% safranine O/sodium acetate buffer. Subsequently, the severity of cartilage deterioration was evaluated using the 0–6 scoring system recommended by the Osteoarthritis Research Society International (OARSI) [17] (Table 1) and observed under an Olympus BX51 light microscope. Images were obtained using an Olympus DP72 digital camera (Olympus, Tokyo, Japan).

**Table 1.** Osteoarthritis Research Society International (OARSI) score

Grade	Osteoarthritic damage
0	Normal
0.5	Loss of Safranin-O without structural changes
1	Small fibrillations without loss of cartilage
2	Vertical clefts down to the layer immediately below the superficial layer and some loss of surface lamina
3	Verical clefts/erosion to the calcified cartilage extending to < 25% of the articular surface
4	Verical clefts/erosion to the calcified cartilage extending to 25%-50% of the articular surface
5	Verical clefts/erosion to the calcified cartilage extending to 50%-75% of the articular surface
6	Verical clefts/erosion to the calcified cartilage extending to > 75% of the articular surface

### 2.5 Enzyme-linked immunosorbent assay (ELISA)

The expression patterns of IL-1β, IL-18, and cleaved caspase-1 were detected using ELISA kits (Abcam, Cambridge, MA, USA) [18]. In brief, the samples to be tested (containing antibody) were bound to the antigen, and then the labeled enzyme was bound to this complex to obtain an antigen-antibody-labeled enzyme complex, followed by the addition of the substrate of the enzyme to produce the colored product. The optical density value was subsequently determined using a spectrophotometer.

Additionally, the levels of cellular reactive oxygen species (ROS) were detected using ROS detection kits (Jiancheng Bioengineering Institute, Nanjing, China). In short, the cells were placed in 0.01 moL/L phosphate-buffered saline, centrifuged at 4500 g for 10 min to collect the supernatant (190 μL), and then incubated with dichlorofluorescein diacetate (10 μL, 1 moL/L) at room temperature for 30 min. Afterward, the samples were detected using the fluorescence spectrophotometry method.

### 2.6 Quantitative real-time polymerase chain reaction (qRT-PCR)

Total RNA content was extracted from tissues and cells using the TRIzol reagent (Invitrogen) [19], and the concentration of the extracted RNA was determined with the help of a DU-640 spectrophotometer (Beckman, San Jose, CA, USA). The extracted RNA was reverse-transcribed into cDNA using PrimeScript RT kits (Takara, Tokyo, Japan) as follows: reverse transcription at 37°C for 15 min and reverse transcriptase inactivation at 85°C for 5 s. Real-time fluorescence quantitative PCR was performed on an ABI7500 fluorescence quantitative PCR instrument (Applied Biosystems, Foster City, CA, USA). The relative expression of miR-140-5p and CTSB was calculated using the 2^−ΔΔCt^ method [20], with glyceraldehyde-3-phosphate dehydrogenase (GAPDH) or U6 serving as the internal reference. Each sample was tested 3 times independently. The reaction conditions were as follows: pre-denaturation at 95°C for 10 min, and 40 cycles of denaturation at 95°C for 15 s and annealing at 60°C for 1 min. The real time fluorescence quantitative PCR system comprised of the following: 9.0 μL SYBR mix, 0.5 μL forward primer, 0.5 μL reverse primer, 2.0 μL cDNA template, 8.0 μL RNase dH2O, for a total volume of 20 μL. The primers are shown in Table 2.

**Table 2.** Primer sequences for qRT-PCR

Name of primer	Sequences
miR-140-5p-F	CGCATGGCAGTGGTTTTACCCTA
miR-140-5p-R	ATCCAGTGCAGGGTCCGAGG
CTSB-F	ATGGTGGCTATCCCTCTGGAG
CTSB-R	AGAGGGACAATCATCAGG
GAPDH-F	AGTTGTGGGCAAACACACTCA
GAPDH-R	TGTAGACCATGTAGGTCA
U6-F	CTCGCTTCGGCAGCACATATACT
U6-R	ACGCTTCACGAATTTGCGTGTC

qRT-PCR: quantitative real-time polymerase chain reaction; miR-140-5p: microRNA-140-5p; CTSB: cathepsin B; GAPDH: glyceraldehyde-3-phosphate dehydrogenase

### 2.7 Western blot

The protein levels in cartilage tissues and cells were determined by referring to methods in previous literature [19]. Briefly, the protein lysates of brain tissues or cell samples were prepared with a radio-immunoprecipitation assay lysis buffer (P1003B, Beyotime, Shanghai, China) and 1% phenylmethylsulfonyl fluoride. Bicinchoninic acid (BCA) kits (Beyotime) were adopted for protein quantification. Subsequently, the proteins (15–50 μg) were separated with 4–20% sodium dodecyl sulfate polyacrylamide gel electrophoresis and transferred onto polyvinylidene difluoride membranes with a pore size of 0.45 μm or 0.22 μm. Next, the membranes were blocked with 5% skim milk for 1 h and cultured with the primary antibodies gasdermin D (GSDMD)-N (dilution ratio of 1:1000, DF13758, Affinity Biosciences, USA), CTSB (dilution ratio of 1:1000, ab214428, Abcam), NLRP3 (dilution ratio of 1:1000, ab263899, Abcam), and GAPDH (dilution ratio of 1:10,000, ab181602, Abcam) at 4°C overnight. Afterward, the membranes were cultured with the secondary antibody anti-rabbit IgG (dilution ratio of 1:1000, ab205718, Abcam) for 1 h, and then visualized using an enhanced chemiluminescence reagent (#34,080, Thermo Fisher Scientific Inc., Waltham, MA, USA). The protein blotting was analyzed using the ImageQuant LAS 4000 (General Electric Company, Schenectady, NY, USA).

### 2.8 Immunohistochemical (IHC) staining

The expression patterns of cleaved caspase-1 in cartilage tissues were detected by referring to methods in previous literature [19]. Briefly, the tissues were fixed with 4% paraformaldehyde, paraffin-embedded, sectioned continuously (at 5 μm), baked at 58°C for 18 h, and dewaxed with xylene. Next, the sections were treated with 0.01 mol/L citric acid buffer at 95°C (5 min × 2 times) for antigen retrieval, incubated with 3% H_2_O_2_ for 15 min at room temperature, and then incubated with the primary antibody (cleaved caspase-1, dilution ratio of 1:1000, PA5-99,390, Thermo Fisher Scientific) at 4°C overnight and the secondary antibody at room temperature for 30 min. Afterward, the sections were developed with 2,4-diaminobutyric acid for 5 min, rinsed under running water to terminate the reaction, counterstained with hematoxylin, and sealed with resin. Subsequently, 9 visual fields were randomly selected from each sample to calculate the percentage of positive cells.

### 2.9 Cell counting kit-8 (CCK-8) assay

The proliferation ability of chondrocytes was measured by referring to methods in previous literature [21]. Briefly, the cells were seeded in 96-well plates (3000 cells per well) and cultured at 37°C. After incubation for 0 h, 24 h, 48 h, and 72 h, each well was added with 10 μL CCK-8 reagent (Beyotime) and incubated for 4-h at 37°C. Afterward, the absorbance at a wavelength of 450 nm was detected and a cell proliferation curve was drawn.

### 2.10 Dual-luciferase assay

The binding site of miR-140-5p and CTSB was analyzed with the help of the StarBase website (http://starbase.sysu.edu.cn/) [22]. Subsequently, the binding sequence and mutant sequence were cloned into the luciferase vector pGL3 (Promega, Madison, WI, USA) to construct the wild-type (CTSB-WT) and mutant-type (CTSB-MUT) luciferase plasmids. Next, 293 T cells (American Type Culture Collection, Manassas, VA, USA) were seeded in 6-well plates (2 × 10^5^ cells/well) and cultured for 24 h. Next, the constructed luciferase vectors were co-transfected with mimic NC or miR-140-5p mimic (Genechem) (miRNA mimic 100 nM) into 293 T cells using Lipofectamine 2000 (11,668–019, Invitrogen). The luciferase activity was evaluated using Dual-Lucy assay kits (Solarbio, Beijing, China) after 24 h. Each cell experiment was repeated 3 times independently to obtain the mean value.

### 2.11 Calcein-AM/propidium iodide (PI) staining

The cells were seeded in 24-well plates (2 × 10^4^ cells/well), cultured for 4 h, and then stained using Calcium-AM/PI kits (Dojindo, Shanghai, China) [23]. Briefly, the samples were treated with 5 μL Calcein-AM (2 μM) and 5 μL PI (2 μM) at 37°C in conditions void of light for 30 min, and then analyzed under a fluorescence microscope.

### 2.12 Co-immunoprecipitation (Co-IP) assay

Co-IP assay were performed by referring to methods in previous literature [24] to confirm the protein-protein binding. Following the addition of protease inhibitor mixture and phosphatase inhibitor mixture (Santa Cruz Biotechnology, Texas, USA), the mouse knee cartilage tissue lysate was produced. Next, the total protein of the lysate was determined using a Genesys 10 UV-Vis spectrophotometer (Thermo Fisher Scientific) and Pierce BCA protein analysis kits (Thermo Science, Waltham, MA, USA). After diluting the sample with lysate to the same concentration, 40 μL protein was collected and used as the Input. The mouse cartilage tissue protein was then treated with the Pierce Co-IP kit (Thermo Fisher Scientific). The experiment was performed in accordance with the manufacturer’s instructions as follows: 10 μL of monoclonal CTSB (1:1000, ab214428, Abcam) was co-incubated with the resin and covalently coupled, and the control group was added with 10 μL anti-IgG (dilution ratio of 1:1000, ab205718, Abcam). The antibody-coupled resin was cultured with 200 mL mouse cartilage tissue protein lysate at 4°C overnight, and the resin was rinsed. Afterward, the protein complex bound to the antibody was eluted, followed by Western blot analysis.

### 2.13 Statistical analysis

Statistical analyses were performed using the SPSS 21.0 software (SPSS Inc, Chicago, IL, USA) and GraphPad Prism 6.0 software (GraphPad Software Inc., San Diego, CA, USA). The Kolmogorov-Smirnov test was adopted to ensure that the data were in compliance with normal distribution. Measurement data were presented as mean ± standard deviation. One-way analysis of variance (ANOVA) was employed for comparisons among multiple groups, followed by Tukey’s multiple comparisons test. The *p* value was obtained by a two-tailed test, and a value of *p* < 0.05 was regarded statistically significant.

## 3. Results

In this study, we explored the biological role and molecular mechanism of miR-140-5p in OA. Our data showed that miR-140-5p was poorly expressed in OA. Upregulation of miR-140-5p alleviated cartilage injury and reduced chondrocyte pyroptosis caused by OA. miR-140-5p inhibited the binding of CTSB to NLRP3 by targeting the expression of CTSB. In conclusion, our study showed the role of miR-140-5p in OA chondrocyte injury via the CTSB/NLRP3 axis, which meant that targeting these molecules may be a new method for the treatment of OA.

### 3.1. Establishment of the murine model of OA

**Figure 1.**
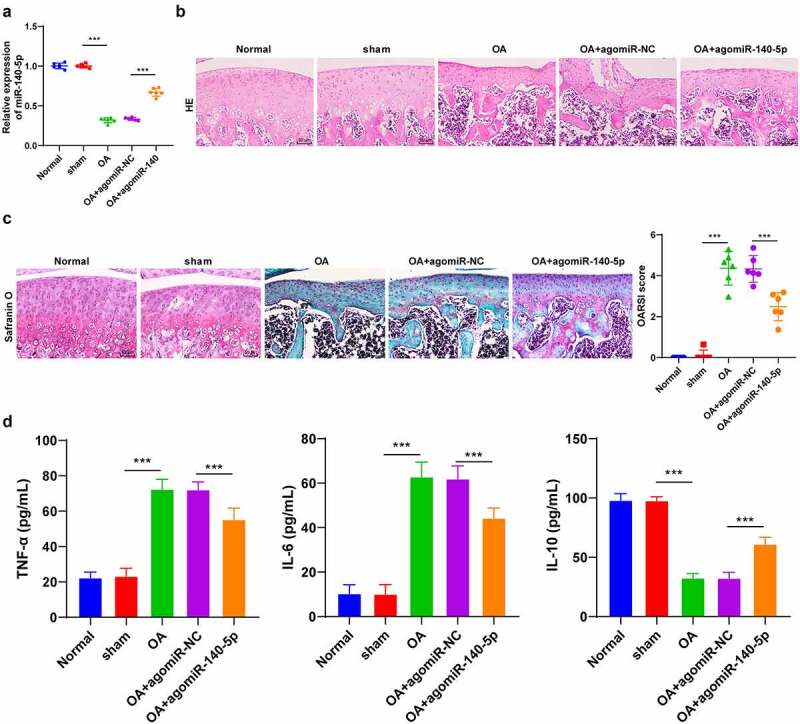
OA induction promoted cartilage tissue degradation. the OA model was established by operation in BALB/c mice. A: miR-140-5p expression was detected using qRT-PCR. B: representative images of hematoxylin and eosin staining. C: safranin O staining. D: inflammatory factors (TNF-α, IL-6, and IL-10) were detected using ELISA kits. N = 6. data in panels A and C are enumeration data; data in panel D is measurement data and expressed as mean ± standard deviation. data were analyzed using one-way ANOVA, followed by Tukey’s multiple comparisons test, ***p < 0.001. OA: Osteoarthritis

Firstly, to explore the mechanism of miR-140-5p in OA cartilage injury, we established murine models of OA with the help of BALB/c mice. It was found that OA mice presented with notably decreased miR-140-5p expression levels in cartilage tissues (*p* < 0.001; Figure 1(a)), in addition to enhanced cartilage tissue degradation and elevated OARSI score (*p* < 0.001; Figure 1(b-c)). Meanwhile, following injection of agomiR-140-5p to up-regulate miR-140-5p expression (*p* < 0.001; Figure 1(a)), we observed that the cartilage tissue degradation was reduced and OARSI score was decreased in OA mice (*p* < 0.001; Figure 1(b-c)). Furthermore, we detected the levels of inflammatory factors in OA mice using ELISA kits, and the results demonstrated that the pro-inflammatory factors TNF-α and IL-6 were both significantly elevated, while the anti-inflammatory factor IL-10 was decreased in the cartilage tissues of OA mice, whereas over-expression of miR-140-5p brought about the opposite trends (*p* < 0.001; Figure 1(d)). Altogether, these findings indicated that OA induction promoted cartilage degradation, inhibited miR-140-5p expression, and enhanced the secretion of inflammatory factors and reduced the secretion of anti-inflammatory factors.

### 3.2. Up-regulation of miR-140-5p alleviated chondrocyte pyroptosis in OA mice

**Figure 2.**
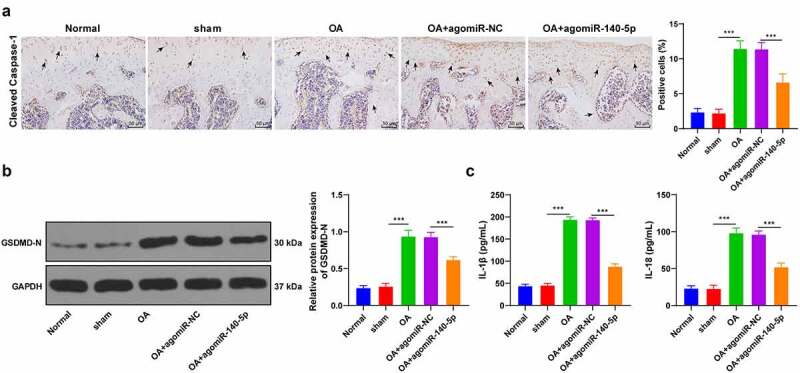
Upregulation of miR-140-5p alleviated chondrocyte pyroptosis in OA mice. the OA mice were injected with agomiR-140-5p, with agomiR-NC as the control. A: cleaved caspase-1 positive expression was detected using immunohistochemical staining; the arrow indicates positive staining. B: GSDMD-N protein level was detected using western blot. C: IL-1β and IL-18 levels were detected using ELISA kits. N = 6. the measurement data are expressed as mean ± standard deviation and analyzed using one-way ANOVA, followed by Tukey’s multiple comparisons test, ***p < 0.001

Pyroptosis is closely associated with cartilage degradation during the progression of OA [2]. Accordingly, we speculated whether miR-140-5p played a role in OA by regulating chondrocyte pyroptosis. Subsequently, we detected the levels of cleaved caspase-1 in tissues using IHC staining and found that cleaved caspase-1 levels in OA mice were notably higher compared to those in sham-operated mice and normal mice, while up-regulation of miR-140-5p reversed the increase of cleaved caspase-1 (*p* < 0.001; Figure 2(a)). Moreover, GSDMD-N levels were determined detected using Western blot, and the results showed that OA induction up-regulated GSDMD-N levels, while over-expression of miR-140-5p brought about a reduction in GSDMD-N levels (*p* < 0.001; Figure 2(b)). Meanwhile, the results of ELISA illustrated that over-expression of miR-140-5p reversed the increase of pyroptosis-related inflammatory factors (IL-1β and IL-18) (*p* < 0.001; Figure 2(c)). Together, these findings indicated that up-regulation of miR-140-5p protected OA mice against chondrocyte pyroptosis.

### 3.3. Up-regulation of miR-140-5p alleviated LPS-induced chondrocyte pyroptosis in vitro

**Figure 3.**
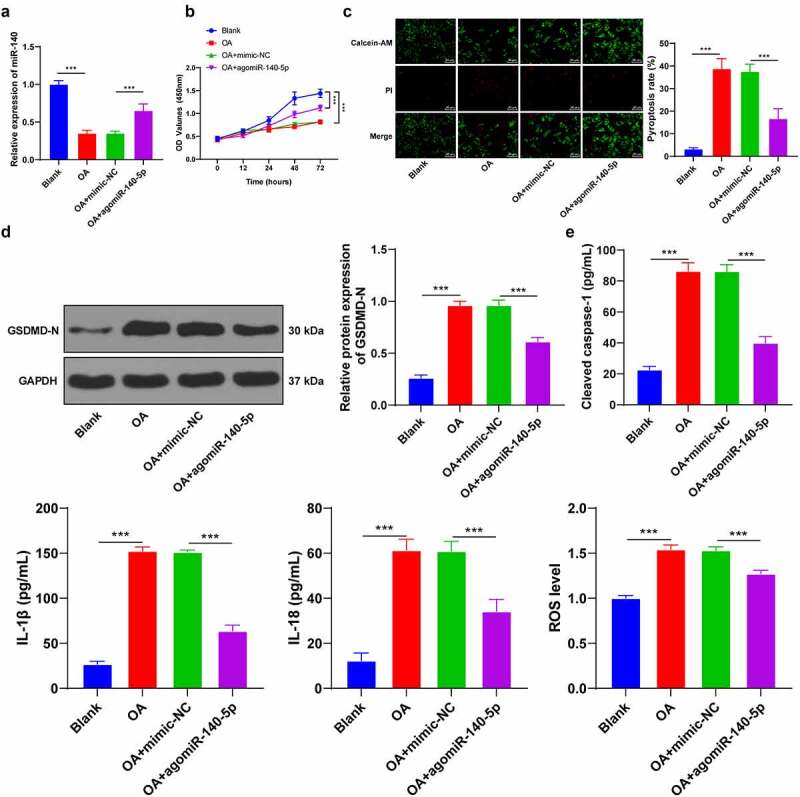
Upregulation of miR-140-5p alleviated LPS-induced chondrocyte pyroptosis *in vitro*. mouse chondrocytes were isolated and cultured *in vitro*. the cell model of OA was established by LPS induction and the model cells were transfected with miR-140-5p-mimic, with miR-NC as the control. A: miR-140-5p expression in cells was detected using qRT-PCR. B: cell viability was detected using CCK-8 assay. C: Pyroptosis was measured using calcein-AM/PI staining. D: GSDMD-N protein level was detected using western blot. E: cleaved caspase-1, IL-1β, IL-18, and ROS levels were detected using the kits. the cell experiment was repeated 3 times independently. the measurement data are expressed as mean ± standard deviation. data in panels A/C/D/E were analyzed using one-way ANOVA, and data in panel B were analyzed using two-way ANOVA, followed by Tukey’s multiple comparisons test, ***p < 0. 001

To further validate the regulatory mechanism of miR-140-5p in OA, we isolated chondrocytes from mice and established cell models of OA by means of LPS induction. It was subsequently observed that miR-140-5p was poorly-expressed in OA chondrocytes, while miR-140-5p mimic augmented the miR-140-5p expression (*p* < 0.001; Figure 3(a)). Meanwhile, LPS induction reduced cell viability and enhanced pyroptosis, as evidenced by increased GSDMD-N fragments, enhanced ROS and cleaved caspase-1, and elevated pyroptosis-related inflammatory factors, whereas up-regulation of miR-140-5p partially reversed the aforementioned index changes (all *p* < 0.001; Figure 3(b-e)). Overall, these findings suggested that up-regulation of miR-140-5p alleviated LPS-induced chondrocyte pyroptosis.

### 3.4. miR-140-5p targeted CTSB transcription

**Figure 4.**
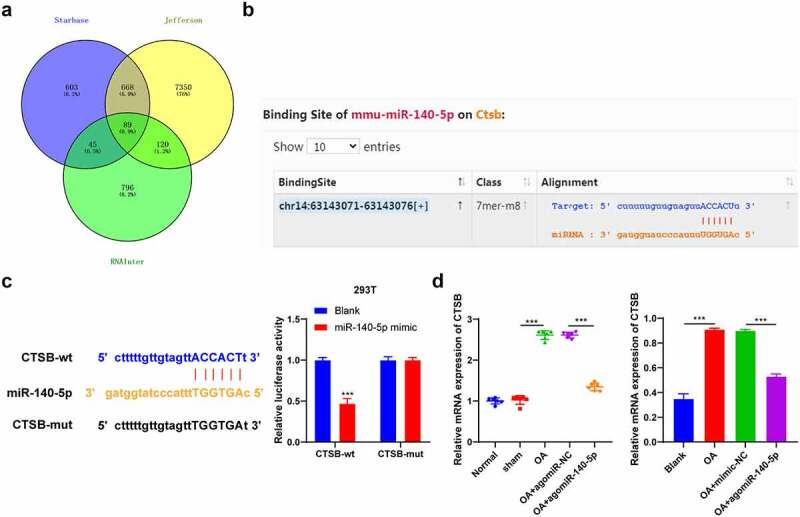
miR-140-5p targeted CTSB transcription. A: the downstream target genes of miR-140-5p were predicted through starbase (http://starbase.sysu.edu.cn/), jefferson (https://cm.jefferson.edu/rna22/precomputed/), and RNAInter (http://www.rna-society.org/raid/search.html) websites. B: the binding site between miR-140-5p and CTSB was predicted through starbase website. C: the binding relationship between miR-140-5p and CTSB was verified using dual-luciferase assay. D: CTSB expression was detected using qRT-PCR. the cell experiment was repeated 3 times independently. data in panel B (left) are enumeration data; data in panels B (right) and C are measurement data and expressed as mean ± standard deviation. data in panel C were analyzed using two-way ANOVA, and data in panel D were analyzed using one-way ANOVA, followed by Tukey’s multiple comparisons test, ***p < 0. 001

To further explore the downstream mechanism of miR-140-5p in OA, we predicted the downstream target genes of miR-140-5p with the help of the Starbase (http://starbase.sysu.edu.cn/), Jefferson (https://cm.jefferson.edu/rna22/Precomputed/), and RNAInter (http://www.rna-society.org/raid/search.html) databases and obtained the intersection (Figure 4(a)), wherein which we focused our efforts on CTSB. A number of studies have elucidated that CTSB participates in the pro-inflammatory mechanism of cells [25,26]. As a result, we speculated that miR-140-5p played a role in OA by targeting CTSB. Results from the Starbase website provided the binding site between miR-140-5p and CTSB (Figure 4(b)), whereas a dual-luciferase assay performed in 293 T cells verified the binding relationship between miR-140-5p and CTSB (*p* < 0.001; Figure 4(c)). Furthermore, the results of qRT-PCR results showed that CTSB mRNA expression levels were elevated in OA mice and cells, while up-regulation of miR-140-5p reversed the increase of CTSB (*p* < 0.001; Figure 4(d)). Altogether, these findings indicated that miR-140-5p negatively-regulated CTSB transcription.

### 3.5. Up-regulation of CTSB annulled the inhibitory effect of miR-140-5p over-expression on chondrocyte pyroptosis

**Figure 5.**
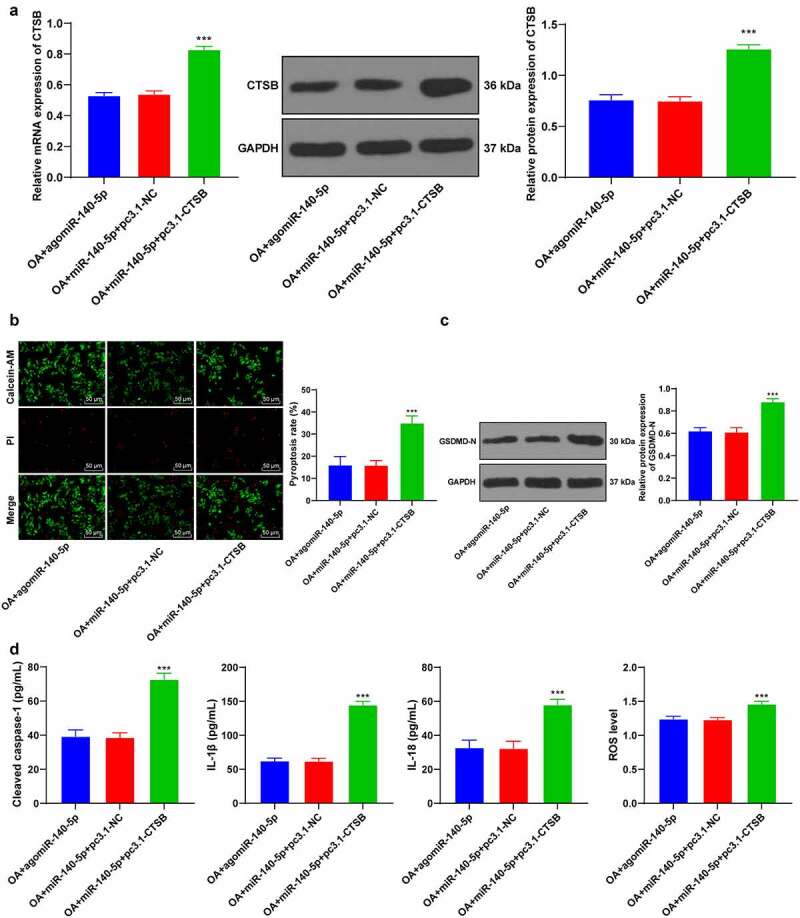
Upregulation of CTSB attenuated the inhibitory effect of miR-140-5p overexpression on chondrocyte pyroptosis. the OA cells with high expression of miR-140-5p were transfected with pcDNA3.1-CTSB, with pcDNA3.1-NC as the control. A: CTSB expression was detected using qRT-PCR and western blot. B: Pyroptosis was measured using calcein-AM/PI staining. C: GSDMD-N protein level was detected using western blot. D: cleaved caspase-1, IL-1β, IL-18, and ROS levels were detected using the kits. the cell experiment was repeated 3 times independently. the measurement data are expressed as mean ± standard deviation and analyzed using one-way ANOVA, followed by Tukey’s multiple comparisons test, ***p < 0. 001

To further verify the regulatory mechanism of miR-140-5p/CTSB, we performed a series of functional rescue experiments. We up-regulated the expression of CTSB in OA chondrocytes with high expressions of miR-140-5p (*p* < 0.001; Figure 5(a)), which revealed that up-regulation of CTSB attenuated the inhibitory effect of miR-140-5p over-expression on chondrocyte pyroptosis (all *p* < 0.001; Figure 5(b-d)). Overall, these findings suggested that up-regulation of CTSB partly-reversed the inhibitory effect of miR-140-5p over-expression on chondrocyte pyroptosis in OA.

### 3.6. CTSB bound to NLRP3 and promoted the expression of NLRP3

**Figure 6.**
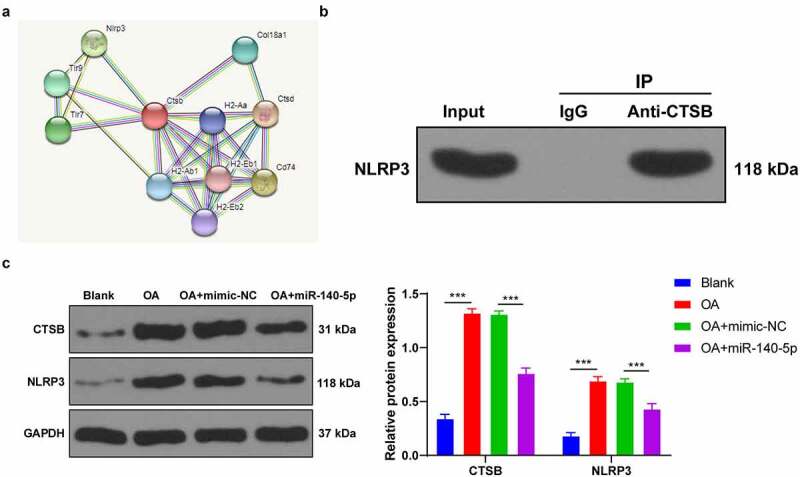
CTSB bound to NLRP3 and promoted the expression of NLRP3. A: protein interaction network of CTSB was predicted through STRING website (https://string-db.org/cgi/input.pl?sessionId=MzVoWhbW9Q40&input_page_show_search=on). B: the binding relationship between CTSB and NLRP3 was verified using Co-IP assay. C: CTSB and NLRP3 protein levels were detected using western blot. the cell experiment was repeated 3 times independently. the measurement data are expressed as mean ± standard deviation and analyzed using two-way ANOVA, followed by Tukey’s multiple comparisons test, ***p < 0. 001

To further elucidate the regulatory mechanism of CTSB in pyroptosis, we predicted the protein interaction relationship of CTSB using the STRING website (https://string-db.org/cgi/input.pl?sessionId=MzVoWhbW9Q40&input_page_show_search=on), which revealed the presence of a binding relationship between CTSB and pyroptosis-related NLRP3 inflammasome (Figure 6(a)). Thereafter, we speculated that CTSB played a role in OA by interacting with NLRP3. The results of Co-IP assay verified the binding relationship between CTSB and NLRP3 in chondrocytes (Figure 6(b)). Furthermore, CTSB and NLRP3 protein levels in each group of cells were detected using Western blot, and we found that CTSB and NLRP3 protein levels were both increased in OA chondrocytes, while up-regulation of miR-140-5p reduced CTSB and NLRP3 protein levels (all *p* < 0.001; Figure 6(c)). Altogether, these findings indicated that up-regulation of miR-140-5p inhibited CTSB protein levels in OA chondrocytes, and thus inhibited the expression of NLRP3.

### 3.7. Up-regulation of miR-140-5p reduced chondrocyte pyroptosis by inhibiting the binding of CTSB/NLRP3

**Figure 7.**
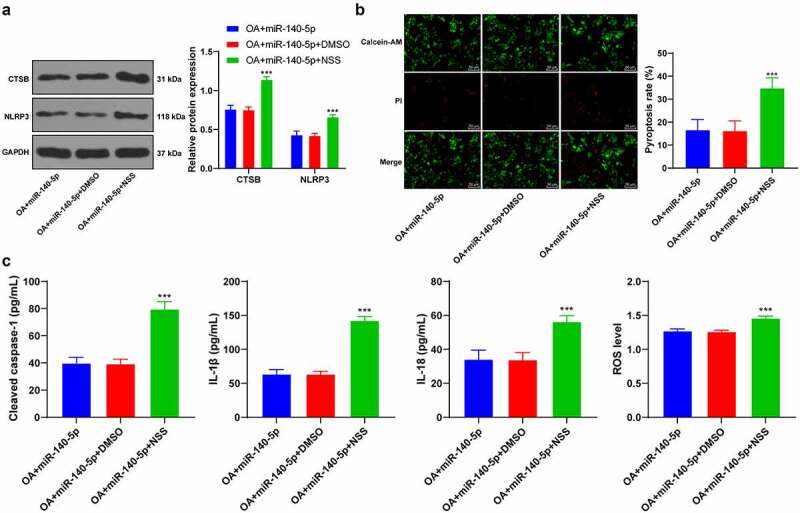
Upregulation of miR-140-5p reduced chondrocyte pyroptosis by inhibiting the binding of CTSB/NLRP3. the OA cells with high expression of miR-140-5p were treated with Nigericin sodium salt (NSS), an agonist of NLRP3, with DMSO as the control. A: CTSB and NLRP3 protein levels were detected using western blot. B: Pyroptosis was measured using calcein-AM/PI staining. C: cleaved caspase-1, IL-1β, IL-18, and ROS levels were detected using the kits. the cell experiment was repeated 3 times independently. The measurement data are expressed as mean ± standard deviation. data in panel A were analyzed using two-way ANOVA, and data in panels B/C were analyzed using one-way ANOVA, followed by Tukey’s multiple comparisons test, ***p < 0. 001

Lastly, to validate that miR-140-5p played a role in OA cells by regulating the binding of CTSB/NLRP3, we activated NLRP3 inflammasome in OA cells with high expressions of miR-140-5p using NSS, an agonist of NLRP3 (Figure 7(a)). It was observed that NSS treatment activated NLRP3 inflammasome, enhanced chondrocyte pyroptosis, and increased pyroptosis-related inflammatory factors (all *p* < 0.001; Figure 7(b-c)). Collectively, these findings suggested that miR-140-5p alleviated chondrocyte pyroptosis in OA by inhibiting the CTSB/NLRP3 axis.

## 4. Discussion

**Figure 8.**
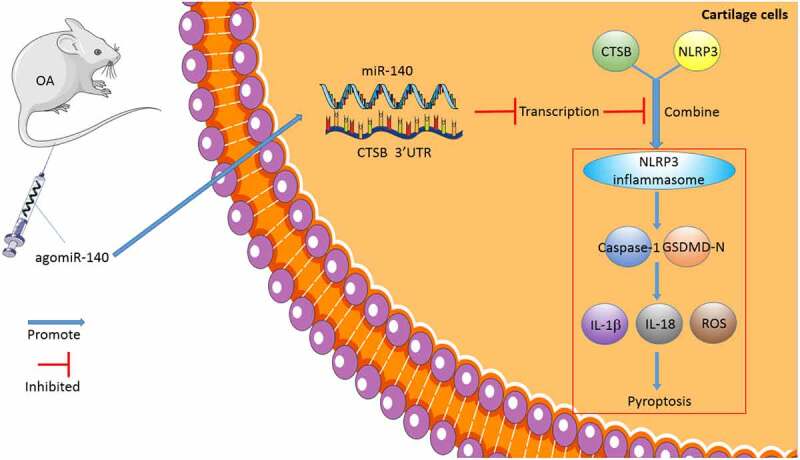
The mechanism of miR-140-5p affecting chondrocyte pyroptosis and repairing OA. miR-140-5p was overexpressed in the murine model of OA by agomiR-140-5p injection, thus inhibiting CTSB transcription, further repressing the binding of CTSB and NLRP3, reducing chondrocyte pyroptosis, and alleviating cartilage injury in OA

OA is one of the leading causes of pain, disability, and socioeconomic cost across the world, with its ever-increasing prevalence being very alarming [27]. Interestingly, the hard-done work of our peers suggests that miR-140-5p is specifically-expressed in OA, highlighting miR-140-5p as a novel target for gene therapy [10]. In an effort to expand our knowledge on the same, the current study set out to elucidate the underlying mechanism of miR-140-5p in OA, and the obtained findings revealed that miR-140-5p alleviated OA cartilage injury by repressing chondrocyte pyroptosis *via* the CTSB/NLRP3 axis (Figure 8).

OA is well-recognized as a ‘wear and tear’ disease associated with inflammation and aging; more specifically, synovial inflammation can lead to enhanced extracellular matrix degradation in cartilage, resulting in the destruction and degeneration of articular cartilage [28]. miR-140-5p has been documented to exhibit differential expressions in OA [29]. Nevertheless, the specific mechanism of miR-140-5p and its potential influence on OA cartilage injury remains to be fully clarified. During the course of our study, we established murine models of OA and found that OA mice exhibited significantly diminished miR-140-5p expression in cartilage tissues, in addition to enhanced cartilage tissue degradation and augmented inflammatory response. Subsequently, we treated the OA mice with agomiR-140-5p to up-regulate miR-140-5p expression, and we observed that miR-140-5p over-expression could alleviate the aforementioned cartilage degradation and inflammatory responses. In line with our findings, a prior study has revealed that miR-140-5p is essential for *in vitro* chondrogenesis whilst being down-regulated in osteoarthritic cartilages [30]. Moreover, miR-140-5p is indicated to protect chondrocytes from inflammatory injury during the pathogenesis of OA [31], whereas another prior investigation has uncovered that inhibition of miR-140-5p promotes the cartilage matrix degradation in OA cells [32]. Altogether, these findings and evidence indicate that OA induction inhibits miR-140-5p expression, while over-expression of miR-140-5p alleviates cartilage degradation and inflammation.

Chondrocyte death is a common occurrence in the course of degenerative joint diseases, which consequently affects tissue maintenance and functionality and underscores the role of cell death/survival in OA pathogenesis [33]. On a similar note, pyroptosis represents an inflammatory form of caspase-1-dependent cell death [34], whereas inflammasome-associated caspase-1 is known to mediate the maturation of pro-inflammatory cytokines IL-1β and IL-18 and activate the pore-forming protein GSDMD [35]. It is also noteworthy that the prior study has indicated the presence of a potential relationship between pyroptosis and OA risk factors, as well as pyroptosis’ s contribution to cartilage degeneration, synovitis, and OA pain [2]. In our study, we came across increased levels of pyroptosis-related proteins (cleaved caspase-1 and GSDMD-N) and inflammatory factors (IL-1β and IL-18) in OA mice, which confirmed the occurrence of chondrocyte pyroptosis in the pathological process of OA, meanwhile, the chondrocyte pyroptosis of OA mice could be alleviated by miR-140-5p over-expression treatment. Furthermore, we isolated chondrocytes from mice and established a cell model of OA by LPS induction. Consistent with our *in vivo* experimental results, we observed that miR-140-5p was poorly-expressed in OA chondrocytes, whereas up-regulation of miR-140-5p reduced LPS-induced chondrocyte pyroptosis. A prior investigation has suggested that miR-140-5p retards OA progression by suppressing chondrocyte apoptosis, which is in accordance with our findings [9]. To the best of our knowledge, our study is the first-of-its-kind to demonstrate that miR-140-5p protects OA mice against cartilage injury by inhibiting chondrocyte pyroptosis.

Thereafter, we shifted our efforts to uncovering the downstream mechanism of miR-140-5p in chondrocyte pyroptosis. Subsequently, the target genes of miR-140-5p were predicted with the help of various online websites, and the CTSB gene was selected for further exploration. CTSB is already implicated in OA pathophysiology due to its increased expressions in pro-inflammatory conditions, such that monitoring CTSB activity has been previously indicated to assist the assessment of OA severity [14]. Moreover, our findings demonstrated that CTSB was highly-expressed in OA mice and chondrocytes, while the results of a dual-luciferase assay verified the binding relationship between miR-140-5p and CTSB. In addition, we carried our functional rescue experiments, and discovered that up-regulation of CTSB weakened the inhibitory effect of miR-140-5p on chondrocyte pyroptosis. Similarly, the study performed by *Chen et al*. has suggested that CTSB is leaked through lysosomal membrane permeabilization into the cytosol and induces pyroptosis [36]. Furthermore, As2O3-induced pyroptosis is dependent upon CTSB-mediated inflammasome activation, while inhibition of CTSB is known to reduce inflammasome activation and pyroptosis [26]. Additionally, we predicted the protein interaction relationship of CTSB through the STRING database, and came across a binding relationship between CTSB and NLRP3. Notably, NLRP3 is a well-characterized inflammasome that functions as a molecular switch modulating inflammatory response [37]. What’s more, a number of emerging studies have implied that NLRP3 participates in the inflammatory response of various diseases, including OA [38–40]. Findings of our Co-IP assay verified the binding relationship between CTSB and NLRP3 in chondrocytes, whereas CTSB and NLRP3 protein levels were both increased in OA chondrocytes, such that up-regulation of miR-140-5p diminished the CTSB and NLRP3 protein levels, which suggested that up-regulation of miR-140-5p decreased CTSB protein levels in OA chondrocytes and thereby suppressed the expression of NLRP3. Consistently, another prior study has indicated that NLRP3 inflammasome is highly-implicated in fibroblast-like synoviocyte inflammation and pyroptosis, and inhibition of NLRP3 leads to a significant reduction of pyroptosis-related cytokines [5]. Furthermore, NLRP3 inflammasome activation using an NLRP3 agonist NSS enhanced chondrocyte pyroptosis and increased pyroptosis-related inflammatory factors. NLRP3-inflammasome further leads to synovial inflammation by activating toll-like receptors and NF-κB signaling, thus deteriorating the development of OA [41]. Altogether, these findings and evidence indicate miR-140-5p reduces chondrocyte pyroptosis in OA by inhibiting the CTSB/NLRP3 axis.

## 5. Conclusions

To sum up, our findings indicated that miR-140-5p suppressed the binding of CTSB and NLRP3 protein by targeting CTSB, thus inhibiting NLRP3 inflammasome and improving OA chondrocyte pyroptosis. However, our study merely highlighted that miR-140-5p can be adopted as a therapeutic target for OA in animal and cell models, but we did not verify whether the miR-140-5p/CTSB/NLRP3 axis can serve as a clinical therapeutic target for OA. Moreover, NLRP3 inflammasome, as a classic inflammatory factor, can be regulated by a plethora of proteins and pathways in various diseases, thus it requires further exploration whether miR-140-5p can regulate NLRP3 inflammasome through other proteins and pathways in OA. We shall explore the regulation mechanism of the miR-140-5p/CTSB/NLRP3 axis in osteoarthritis clinically in our future endeavors.

## Data Availability

The data that support this study are available from the corresponding author upon reasonable request.

## Research Highlights

1. miR-140-5p is poorly expressed in osteoarthritis.
2. miR-140-5p overexpression reduces cartilage injury and chondrocyte pyroptosis.
3. miR-140-5p targets CTSB.
4. CTSB binds to NLRP3 and promotes NLRP3 expression.
5. miR-140-5p inhibits chondrocyte pyroptosis via the CTSB/NLRP3 axis.
